# A diagnostic challenge of dual atrioventricular nodal nonreentrant tachycardia revealed by ambulatory electrocardiographic monitoring: a case report

**DOI:** 10.3389/fcvm.2026.1843500

**Published:** 2026-05-08

**Authors:** Zhiqin Ma, Jianzhou Tian, Ping Yuan, Jun Shen

**Affiliations:** Department of Cardiology, Renmin Hospital, Hubei University of Medicine, Shiyan, Hubei, China

**Keywords:** case report, dual atrioventricular nodal nonreentrant tachycardia, dual AV nodal physiology, electrocardiogram, Holter monitoring

## Abstract

**Background:**

Dual atrioventricular nodal nonreentrant tachycardia (DAVNNT) is an uncommon arrhythmia caused by simultaneous antegrade conduction of a single atrial impulse through fast and slow atrioventricular nodal pathways, resulting in double ventricular activation without a reentrant circuit. Because its electrocardiographic manifestations are variable and may mimic other supraventricular arrhythmias, DAVNNT is frequently overlooked.

**Case summary:**

A 57-year-old woman was admitted with cough and chest tightness lasting for more than 1 month. During hospitalization, she developed paroxysmal palpitations and exertional chest tightness. Coronary computed tomography angiography showed no obvious stenosis, and cardiac biomarkers were unremarkable. Initial electrocardiograms suggested sinus rhythm with junctional or atrial ectopic activity. However, 24-hour Holter monitoring demonstrated the absence of a strict 1:1 relationship between P waves and QRS complexes, recurrent short and long PR intervals, fast- and slow-pathway Wenckebach conduction, and 1:2 atrioventricular conduction. These findings were highly suggestive of DAVNNT. The patient was referred for further electrophysiological evaluation.

**Conclusion:**

This case highlights the diagnostic value of careful surface ECG and Holter analysis in recognizing DAVNNT before invasive electrophysiological confirmation becomes available. A diagnosis highly suggestive of DAVNNT should be considered when organized atrial activity is associated with more ventricular activations than atrial depolarizations and when fixed short and long PR intervals recur during ambulatory monitoring.

## Introduction

Dual atrioventricular nodal nonreentrant tachycardia (DAVNNT) is a rare manifestation of dual atrioventricular nodal physiology in which one atrial impulse conducts simultaneously over both the fast and slow pathways, producing two ventricular responses without formation of a reentrant circuit. Recognition on routine electrocardiography may be difficult because the rhythm can appear irregular and P waves may be obscured by preceding T waves. As a result, DAVNNT may be misinterpreted as atrial premature beats, atrial tachycardia, junctional ectopy, or other supraventricular tachyarrhythmias (1–4).

Most reported cases emphasize invasive electrophysiological confirmation and catheter ablation (2–4). In contrast, the present case underscores the value of ambulatory electrocardiographic monitoring in raising strong clinical suspicion for DAVNNT in a patient initially admitted to a respiratory department. The case is notable because the arrhythmia was identified against a background of cough, chest tightness, pulmonary nodules, and evaluation for possible tuberculosis, which initially diverted attention away from the heart rhythm disturbance. At the same time, because DAVNNT is fundamentally an electrophysiological diagnosis, the present report is intended to emphasize a noninvasive recognition pattern that is highly suggestive of DAVNNT rather than definitively confirm the diagnosis.

## Case presentation

A 57-year-old woman was admitted with cough and chest tightness for more than 1 month. Her symptoms began approximately 1 month before admission and included intermittent dry cough with small amounts of white sputum, mild chest tightness, shortness of breath, wheezing, poor appetite, fatigue, mild nasal congestion, rhinorrhea, nausea, acid reflux, and dizziness. She denied chest pain, hemoptysis, abdominal pain, diarrhea, and urinary symptoms. Her past medical history was notable for gastritis. She reported no known history of cardiac disease, hypertension, or diabetes mellitus.

On admission, her temperature was 36.5 °C, pulse rate 93 beats/min, respiratory rate 19 breaths/min, blood pressure 110/85 mmHg, and oxygen saturation 98% on room air. Physical examination showed coarse breath sounds without obvious rales. Cardiac examination revealed a regular rhythm without pathological murmurs. No lower-extremity edema was present.

**Figure 1:**
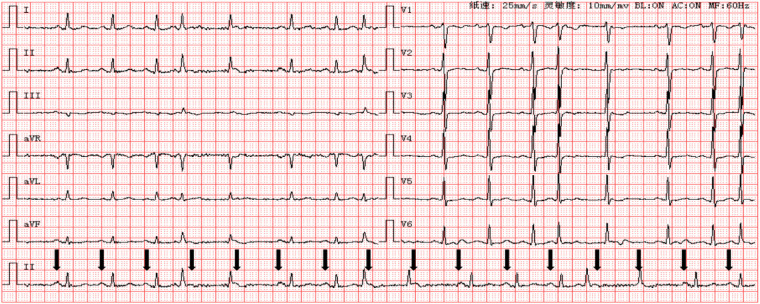
Initial 12-lead electrocardiogram at admission. The initial ECG appeared complex and was not straightforward to classify on first inspection. Early rhythm interpretations favored sinus rhythm with ectopic atrial or junctional activity.

Initial evaluation mainly focused on pulmonary and gastrointestinal causes of the presenting symptoms. Coronary computed tomography angiography performed before admission showed no obvious coronary stenosis. An earlier ECG had shown sinus rhythm, accelerated junctional rhythm, junctional premature beats, and ST-T abnormalities. During the current hospitalization, another ECG was interpreted as sinus rhythm with frequent atrial premature beats, short runs of atrial tachycardia, and T-wave abnormalities. These findings suggested an atrial or junctional mechanism but did not satisfactorily explain the patient's intermittent palpitations and chest tightness (Figure 1).

Laboratory testing showed no significant abnormalities in liver function, kidney function, electrolytes, coagulation profile, brain natriuretic peptide, or troponin. Arterial blood gas analysis showed a pH of 7.420, pO2 of 77.90 mmHg, pCO2 of 34.00 mmHg, and oxygen saturation of 95.60%. Brain CT was unremarkable. Chest CT showed multiple small pulmonary nodules and possible tracheal diverticulum. Pulmonary function testing was essentially normal. Bronchoscopy suggested acute and chronic bronchial inflammation. Repeated sputum smears did not detect acid-fast bacilli, and bronchoalveolar lavage fluid was negative for acid-fast bacilli, fungi, and tuberculosis DNA. Nevertheless, purified protein derivative testing was strongly positive, and the patient later developed erythema nodosum, so tuberculosis remained a clinical consideration.

**Figure 2:**
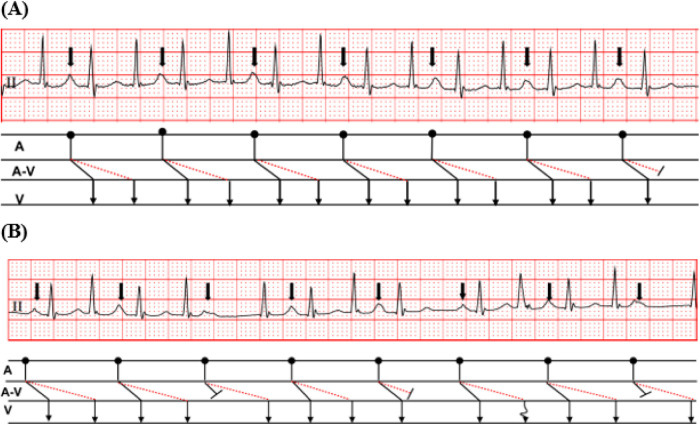
Representative holter strips showing the core diagnostic pattern. **(A)** A representative tracing showing 1:2 atrioventricular conduction, with one atrial depolarization followed by two ventricular responses. **(B)** Recurrent short and long PR intervals in a fixed pattern, supporting conduction over fast and slow AV nodal pathways.

**Figure 3:**
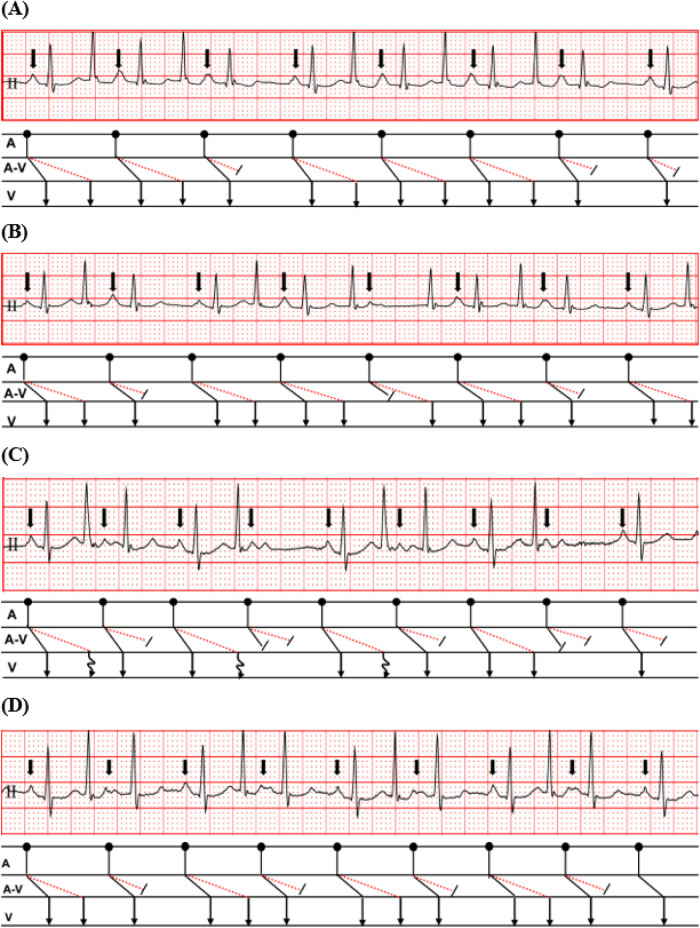
Variable conduction patterns on ambulatory electrocardiographic monitoring. **(A)** Intermittent slow-pathway block with preserved fast-pathway conduction. **(B)** Intermittent fast-pathway block with preserved slow-pathway conduction. **(C)** Alternating fast- and slow-pathway block producing variable ventricular responses. **(D)** Wenckebach-type conduction involving the fast and/or slow pathways.

During hospitalization, the patient experienced paroxysmal palpitations and exertional chest tightness. Twenty-four-hour Holter monitoring demonstrated sinus rhythm with a total of 154,702 beats, an average ventricular rate of 105 beats/min, a maximum ventricular rate of 172 beats/min, and a minimum ventricular rate of 66 beats/min. Importantly, the Holter report suggested dual atrioventricular nodal nonreentrant tachycardia. Characteristic findings included fast- and slow-pathway Wenckebach conduction and 1:2 atrioventricular conduction. These findings indicated dual antegrade AV nodal conduction and were highly suggestive of DAVNNT (Figure 2). Additional representative Holter tracings showed variable conduction behavior, including intermittent fast- or slow-pathway block and Wenckebach-type conduction (Figure 3). Cardiology consultation recommended transesophageal or intracardiac electrophysiological study (EPS) for further clarification. However, EPS was not completed during this admission because the patient was hospitalized primarily for respiratory evaluation.

**Figure 4:**
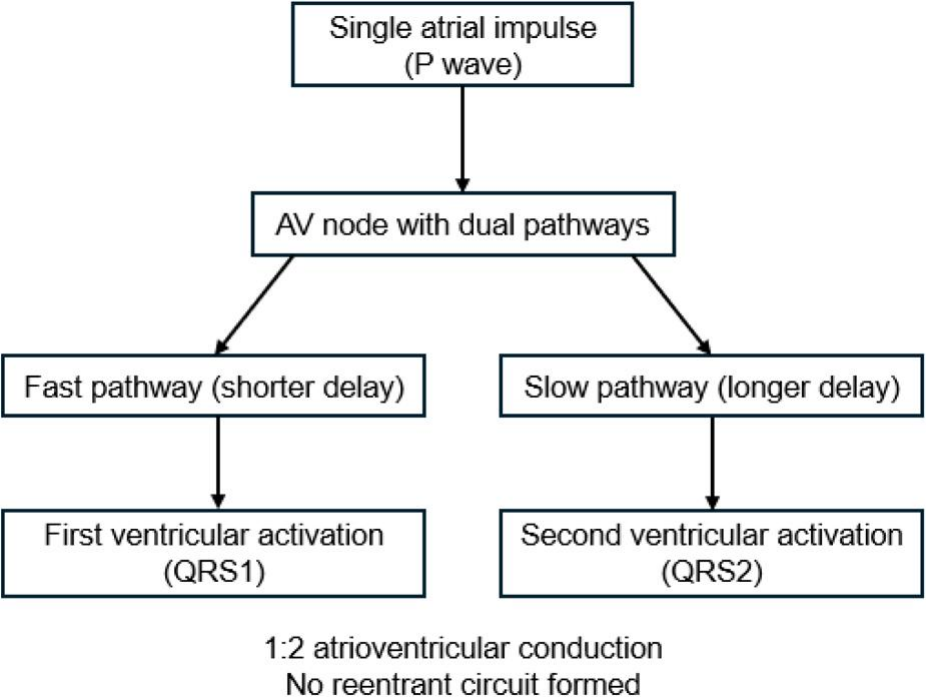
Schematic illustration of the proposed mechanism highly suggestive of dual atrioventricular nodal nonreentrant tachycardia. A single atrial impulse conducts simultaneously over the fast and slow AV nodal pathways, producing two ventricular activations (1:2 conduction) without formation of a reentrant circuit. This schematic is intended for educational illustration of the suspected mechanism.

During the same admission, the patient developed multiple painful erythematous nodules over both lower legs and was diagnosed with erythema nodosum. Autoimmune and tumor-marker testing were negative, and lower-extremity vascular ultrasound showed arterial atherosclerosis without evidence of venous thrombosis. The respiratory team considered a clinical diagnosis of tuberculosis and recommended diagnostic anti-tuberculosis therapy. At discharge, the patient's cough and reflux symptoms had improved, but paroxysmal chest tightness and palpitations persisted intermittently. She was advised to undergo further electrophysiological evaluation as soon as possible. No specific antiarrhythmic drug therapy was initiated during hospitalization, and no clear pharmacological effect on the rhythm disturbance was documented in the medical record. To facilitate understanding of the suspected mechanism, a schematic illustration of dual AV nodal pathway conduction is provided in Figure 4.

## Discussion

This case illustrates the diagnostic challenge of DAVNNT in routine clinical practice. DAVNNT results from simultaneous antegrade conduction of a single atrial impulse through the fast and slow atrioventricular nodal pathways, allowing one P wave to generate two ventricular responses without a reentry circuit. When the difference between fast- and slow-pathway conduction times exceeds the refractory period of the distal conduction system, 1:2 AV conduction can occur (1–3). In theory the mechanism is straightforward, but in practice the surface ECG appearance may be highly variable and misleading.

In the present case, the rhythm was initially interpreted as sinus rhythm with junctional activity, junctional premature beats, frequent atrial premature beats, or short atrial tachycardia. These preliminary interpretations were understandable because DAVNNT may mimic other supraventricular arrhythmias, especially when atrial depolarizations are partly hidden in the T wave and when conduction over the two pathways varies from beat to beat (2–4). The decisive clue came from ambulatory monitoring rather than from a single standard ECG. The Holter recording documented fast- and slow-pathway Wenckebach conduction together with 1:2 AV conduction, findings that strongly support dual antegrade AV nodal physiology.

Another important aspect of this case is the clinical context. The patient was admitted to a respiratory department for cough and chest tightness rather than to a cardiology unit for palpitations. The coexistence of pulmonary nodules, a strongly positive purified protein derivative test, and erythema nodosum complicated the clinical picture and reasonably shifted initial attention toward pulmonary infection or tuberculosis. This background highlights a practical lesson: DAVNNT should remain in the differential diagnosis when patients report paroxysmal palpitations or chest tightness disproportionate to the apparent non-cardiac illness (2, 4).

The differential diagnosis can be expanded further to include atrial tachycardia with variable AV conduction, atypical atrioventricular nodal reentrant tachycardia, high-septal premature ventricular contractions, and concealed accessory pathway–related activity (2–6). Atrial tachycardia with variable AV conduction may produce irregular ventricular responses, but it generally does not account for the repeated pattern of one organized atrial depolarization followed by two ventricular responses with fixed short and long PR intervals. Atypical AVNRT is a reentrant arrhythmia and therefore differs mechanistically from the nonreentrant dual antegrade conduction pattern suspected here. Likewise, high-septal ventricular ectopy or concealed accessory pathway conduction may simulate complex narrow-QRS rhythms, but they do not readily explain the combined presence of recurrent dual PR intervals, fast- and slow-pathway Wenckebach behavior, and 1:2 AV conduction documented on Holter monitoring.

A major limitation of this report is the absence of invasive electrophysiological confirmation at the time of manuscript preparation. Although the Holter findings were highly suggestive of DAVNNT and cardiology consultation recommended transesophageal or intracardiac EPS, intracardiac recordings and ablation outcome were not available in the current record. Therefore, the diagnosis in this report should be interpreted as highly suggestive of DAVNNT rather than definitively confirmed DAVNNT. Nonetheless, the case remains clinically informative because it demonstrates how careful review of surface ECG and Holter tracings can identify the characteristic pattern and appropriately trigger referral for definitive electrophysiological assessment (2–4). Specifically, definitive confirmation would require EPS evidence of simultaneous fast- and slow-pathway conduction, typically with split His-bundle deflections and absence of a reentrant mechanism.

From a clinical management perspective, once DAVNNT is strongly suspected, referral to an electrophysiology service is important because catheter ablation of the slow pathway is generally considered an effective treatment in confirmed cases reported in the literature. Conversely, conservative observation may be reasonable temporarily in clinically stable patients while definitive evaluation is pending. In the present patient, the immediate priority during admission remained the concurrent respiratory work-up.

## Conclusion

This case emphasizes that DAVNNT may be recognized from careful analysis of surface ECG and ambulatory monitoring even before invasive electrophysiological confirmation is available. In patients with palpitations or chest tightness, especially when organized atrial activity is associated with more ventricular activations than atrial depolarizations and recurrent short and long PR intervals, a diagnosis highly suggestive of DAVNNT should be considered in the differential diagnosis. Holter findings such as fast- and slow-pathway Wenckebach conduction and 1:2 atrioventricular conduction provide strong clues and may facilitate timely referral for further electrophysiological evaluation. However, definitive diagnosis still depends on invasive electrophysiological confirmation and exclusion of key mimics.

## Data Availability

The original contributions presented in the study are included in the article/Supplementary Material, further inquiries can be directed to the corresponding author.
